# Mechano-sensitization of mammalian neuronal networks through expression of the bacterial large-conductance mechanosensitive ion channel

**DOI:** 10.1242/jcs.210393

**Published:** 2018-03-01

**Authors:** Alessandro Soloperto, Anna Boccaccio, Andrea Contestabile, Monica Moroni, Grace I. Hallinan, Gemma Palazzolo, John Chad, Katrin Deinhardt, Dario Carugo, Francesco Difato

**Affiliations:** 1 Neuroscience and Brain Technologies Dept., Istituto Italiano di Tecnologia, 16163 Genoa, Italy; 2 Institute of Biophysics, National Research Council of Italy, 16149 Genoa, Italy; 3 Center for Neuroscience and Cognitive Systems, Istituto Italiano di Tecnologia, 38068 Rovereto, Italy; 4 Biological Sciences and Institute for Life Sciences, University of Southampton, SO17 1BJ Southampton, UK; 5 Faculty of Engineering and the Environment, University of Southampton, SO17 1BJ Southampton, UK

**Keywords:** Nanopore engineering, Neuronal mechano-sensitization, Mechanobiology, MscL, Exclusively mechanosensitive ion channel

## Abstract

Development of remote stimulation techniques for neuronal tissues represents a challenging goal. Among the potential methods, mechanical stimuli are the most promising vectors to convey information non-invasively into intact brain tissue. In this context, selective mechano-sensitization of neuronal circuits would pave the way to develop a new cell-type-specific stimulation approach. We report here, for the first time, the development and characterization of mechano-sensitized neuronal networks through the heterologous expression of an engineered bacterial large-conductance mechanosensitive ion channel (MscL). The neuronal functional expression of the MscL was validated through patch-clamp recordings upon application of calibrated suction pressures. Moreover, we verified the effective development of *in-vitro* neuronal networks expressing the engineered MscL in terms of cell survival, number of synaptic puncta and spontaneous network activity. The pure mechanosensitivity of the engineered MscL, with its wide genetic modification library, may represent a versatile tool to further develop a mechano-genetic approach.

This article has an associated First Person interview with the first author of the paper.

## Abstract (precis)

**Summary:** Development and characterization of mechano-sensitized neuronal networks through the heterologous expression of an engineered bacterial large-conductance mechanosensitive ion channel (MscL).

## INTRODUCTION

Neuronal stimulation techniques are essential tools to investigate brain functions and treat neurological diseases (Rogan and Roth, 2011). Current understanding of the mechanisms regulating the physiology of the central nervous system is still limited, thus novel approaches to manipulate the activity of neuronal circuits are required to gain further insights into brain physiology (Panzeri et al., 2017), and to allow the design of alternative and more effective strategies to treat neurological disorders. Established approaches in order to interrogate and dissect the function of neuronal circuits often involve the use of chemical, electrical and/or optical stimulation. Although these methods have allowed important advancements in the field of neuroscience, they all present significant limitations.

Chemical stimulation suffers from poor spatial selectivity and low pharmacokinetic control. The development of a chemogenetic actuator, on the basis of G protein-coupled receptors activated by ad hoc designed synthetic small molecules (DREADDs), provided a cell-type specificity to the chemical stimulation approach (Armbruster et al., 2007), overcoming the selectivity issues. However, DREADD technology still provides a low temporal resolution – with a range of minutes to hours – regarding control the neuronal activity (Whissell et al., 2016).

By contrast, electrical and optical stimulations are paving the way for the development of neuro-prosthetic systems by working at high temporal bandwidth and down to single-cell resolution (Cash and Hochberg, 2015). Their clinical translation is, however, hindered by several practical limitations, including the high degree of surgical complexity and the invasiveness associated with the implantation of stimulation devices (i.e. electrodes and optical fibers). Moreover, related side effects, such as glial scar formation, tissue inflammation, immune responses and performance deterioration of the implanted probes, significantly limit the treatment lifetime (Grill et al., 2009) and complicate the analysis.

Optical stimulation currently represents the most effective strategy to study the physiology of neuronal circuits, as it provides the benefit of contact-free focal stimulation of sub-cellular compartments or cell-type-specific stimulation within a tissue through the selective genetic expression of light-sensitive ion channels (Beltramo et al., 2013).

Drawbacks of this approach are limited penetration into the tissue and phototoxicity accompanied with repeated stimulation. Moreover, both chemogenetic and optogenetic manipulations require genetic modification of the tissue (Jorfi et al., 2015), typically via viral vectors, which limits translation to clinical application. Therefore, within a clinical environment, implantation of electrodes remain the preferred choice to evaluate rehabilitation protocols.

The ideal brain stimulation technology should, thus, avoid implantation of devices, and achieve the wireless remote-modulation of the activity of neuronal circuits. Moreover, it should be safe in the long term, and provide high spatial and temporal control of the stimulus (Tay et al., 2016).

Alternative approaches to the surgical implantation of probes include transcranial electrical, thermal, magnetic and ultrasound stimulation (Fregni and Pascual-Leone, 2007). While transcranial electrical (Grossman et al., 2017) and thermal (Wang and Guo, 2016) stimulations suffer from poor spatial resolution, magnetic and ultrasound fields efficiently propagate across the intact skull bone, and can be focused in small focal volumes at clinically relevant tissue depths (Tyler et al., 2008).

In particular, ultrasound fields provide deeper penetration and improved spatial focusing within dense tissue. Moreover, the use of ultrasound pressure fields as a mean to modulate neuronal activity is attracting considerable interest since ultrasound sources can be miniaturized (Li et al., 2009) and, thus, portable and implantation-free ultrasound stimulation devices could be easily designed. Moreover, the safety of ultrasound waves in biomedical applications has been widely demonstrated, and it is extensively utilized in the clinic for biomedical imaging, rehabilitation physiotherapy, thrombolysis and tumor ablation (Krishna et al., 2017). However, the application of low-intensity ultrasound fields for delicate and reversible alterations in cells and tissues is still in its infancy, due to the limited understanding of the biophysical mechanisms involved (Dalecki, 2004; Tyler, 2011). A similar debate has emerged on the use of magnetic fields, and a unifying theoretical and experimental framework for these forms of stimulation has not been established yet (Meister, 2016). Several models for ultrasound-mediated bioeffects have been proposed, including those based on localized heating, acoustic streaming, intramembrane cavitation (Krasovitski et al., 2011), membrane leaflet separation and modulation of mechanosensitive (MS) ion channels (Tyler, 2011). It is worth noting that direct experimental evidence of ultrasound pressure waves that affect the activity of mechanosensitive ion channels has been provided only recently (Kubanek et al., 2016), thus corroborating the hypothesis that low-intensity ultrasound can, potentially, modulate cellular mechanotransduction pathways (Hertzberg et al., 2010).

In this regard, advances in mechanobiology have led to the discovery, design and application of cellular transduction pathways, as demonstrated in recent studies reporting on the use of mechanosensitive ion channels in order to trigger a cellular response, by using either magnetic (Wheeler et al., 2016) or ultrasound-based (Ibsen et al., 2015) mechanical stimulation. The extraordinary achievements of these studies have laid the foundation of two new research areas, referred to as magnetogenetics and sonogenetics (in addition to the already established optogenetics and chemogenetics). However, most mechanosensitive ion channels, such as TRPV4, display an intrinsic sensitivity to other endogenous stimuli (i.e. voltage, heat, pH, etc.), thus preventing isolated investigation of mechanosensitive responses. Notably, the aforementioned study by Wheeler and colleagues suggests that the overexpression of non-exclusively MS ion channels compromises the physiology of neuronal circuits (Wheeler et al., 2016). Therefore, molecular engineering of these channels is required to render them insensitive to other forms of stimuli.

Mechanotransduction is regarded as one of the evolutionarily oldest signal transduction pathways, and MS channels are one of the most important cellular elements to sense and transduce mechanical forces (Hamill and Martinac, 2001; Martinac, 2014). However, few MS ion channels behave as exclusively mechanosensitive elements, and this list has only recently been updated to include the first mammalian exclusively MS ion channel – the Piezo channel (Coste et al., 2012). Indeed, the first identified exclusively MS ion channel was the bacterial protein known as large-conductance mechanosensitive ion channel (MscL) (Kung et al., 2010; Sukharev et al., 1994). MscL is a homopentameric pore-forming membrane protein that acts as a release valve of cytoplasmic osmolytes when the membrane tension increases (Sawada et al., 2012). The ability to easily isolate large amounts of the MscL from many bacterial strains, and to reconstitute it in a cell-free system, has allowed the detailed characterization of its structure and biophysical properties (Kloda et al., 2008; Martinac et al., 2014; Sukharev et al., 1997). This has facilitated the design and development of new genetically modified variants of the MscL (Maurer and Dougherty, 2003) for potential exploitation in medical and biotechnological applications. Currently, the MscL is the standard biophysical model to study MS channels (Iscla and Blount, 2012), and its large pore diameter of ∼30 Å is considered to be an ideal feature in order to develop triggered nano-valves for controlled drug release (Doerner et al., 2012; Iscla et al., 2013). Notably, thanks to its extensive characterization, the MscL also represents a malleable nano-tool that can be engineered with respect to channel sensitivity (Yoshimura et al., 1999), conductance (Yang et al., 2012) and gating mechanism (Kocer, 2005).

In this paper, we demonstrate the use of the exclusively MS MscL to create mechano-sensitized mammalian neuronal networks and, thus, provide a suitable model to study and further develop the sonogenetic paradigm. We generated an engineered MscL construct for mammalian expression that efficiently localizes to the plasma membrane and, thus, demonstrate the first functional expression of MscLs in primary mammalian neuronal cultures. Moreover, we performed structural and functional characterization of neuronal cells expressing the MscL at both single-cell and network levels. Importantly, we show that the functional expression of the engineered MscLs induces neuronal sensitivity to mechanical stimulation without affecting the physiological development of the neuronal network. Overall, our data demonstrate the development of a mechano-sensitized neuronal network model that reliably allows to investigate, test and calibrate the stimulation of excitable circuits through remotely generated mechanical energy fields.

## RESULTS

### Membrane targeting of the bacterial MscL in primary neuronal cultures

**Fig. 1.**
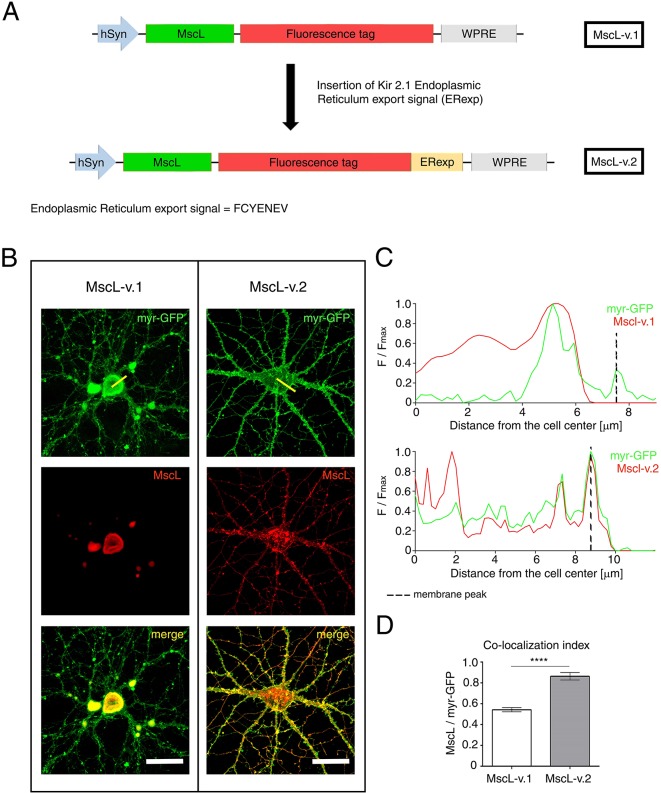
**Membrane targeting of the mammalian-engineered MscL-v.2.** (A) Construct map of the MscL-v.1 (top) and MscL-v.2 (bottom) plasmid in AAV vectors. MscL-v.2 is optimized for expression in mammalian primary neurons. (B) Cortical primary neurons expressing the MscL-v.1 (left) and MscL-v.2 (right) constructs. myr-GFP (green) and MscL fused to tdTomato (red), and their merged fluorescence signals (yellow) are shown to illustrate the reduced aggregation of MscL in the ER, as well as its improved membrane expression after addition of the Kir2.1 ER export signal. Scale bars: 50 µm. Yellow lines represent the cross section by which the fluorescence plot profile was generated. (C) Normalized fluorescence intensity profile of myr-GFP with either MscL-v.1 (top) or MscL-v.2 (bottom). The intensity profiles are extracted along the yellow cross-sectional line shown in B. (D) Colocalization analysis of myr-GFP and either MscL-v.1 or MscL-v.2. The signal of myr-GFP correlates more strongly with that of MscL-v.2 (r=0.86±0.04, *n*=8) than to that of MscL-v.1 (r=0.54±0.02, *n*=11) at the membrane edge. Values are given as mean± s.e.m. The difference between the mean of the two data sets is statistically significant, with a *P* value <0.0001.

In the present work, we established an experimental model of mechano-sensitized neuronal networks. We designed a mammalian expression vector encoding for the bacterial (*Escherichia coli* bacterial strain) MscL fused to tdTomato fluorescent protein under the control of the neuronal-specific synapsin 1 promoter (MscL-v.1, see Fig. 1A).

However, a first functional assessment of MscL-tdTomato expression in primary neuronal cells revealed a significant impairment in the delivery of the heterologous protein to the plasma membrane. In fact, transfected neurons showed large intracellular accumulation and clustering of MscL-tdTomato that, consequently, resulted in low expression on membrane (Fig. 1B, left column panels). We reasoned that the accumulation and clustering of MscL is likely to depend on the lack of a mammalian-specific export signal that prevents protein retention in the endoplasmic reticulum (ER) (Li et al., 2000). Following previous studies that optimized the mammalian expression of optogenetic actuators (Gradinaru et al., 2008), we fused the export signal of Kir2.1 ion channel (MscL-v.2, see Fig. 1A) to the cytoplasmic C-terminus of our MscL-tdTomato protein. The Kir2.1 ER export sequence (FCYENEV) has been extensively studied, and it is known to mediate efficient trafficking and surface expression of the channel (Hofherr, 2005; Stockklausner et al., 2001). Moreover, Kir channel monomers present structural similarities (e.g. two transmembrane domains, a cytoplasmic N- and C-terminus) compared with MscL monomers, thereby suggesting a similar pathway in protein trafficking.

In order to assess the membrane localization of naïve MscLs (MscL-v1) versus MscLs that bear the ER export signal (MscL-v.2), we co-transfected primary neuronal cells in culture with two plasmids: tdTomato-tagged MscL (either MscL-v1 or Mscl-v2) and membrane-targeting myristoylated GFP (myr-GFP). Confocal microscopy examination confirmed enhanced localization of the MscL-v.2 along the neuronal membrane (Fig. 1B, right panels), presumably due to prevention of ER retention and aggregation. In fact, a representative fluorescence intensity profile (along a cross-section line from the center of the cell soma to the plasma membrane; Fig. 1C) of tdTomato-tagged MscL-v.1 (red line), together with the membrane-targeted GFP (green line), shows prominent intracellular localization of MscL-v.1, resulting in the absence of fluorescent colocalization with myr-GFP at the plasma membrane of the cell (vertical dashed lines). By contrast, fluorescence of tdTomato-tagged MscL-v.2 was found to be largely colocalizing with that of myr-GFP, indicating efficient plasma membrane delivery of the channel. Quantitative evaluation of the colocalization index of the two fluorescent proteins by Pearson correlation analysis showed a coefficient of 0.54±0.02 (*n*=11) for the MscL-v.1 construct, indicating no significant co-dependency between the two fluorescence signals, and a coefficient of 0.86±0.04 (*n*=8) for the MscL-v.2 construct, which confirmed a successful increase in membrane expression of the engineered MscL (Fig. 1D).

**Fig. 2.**
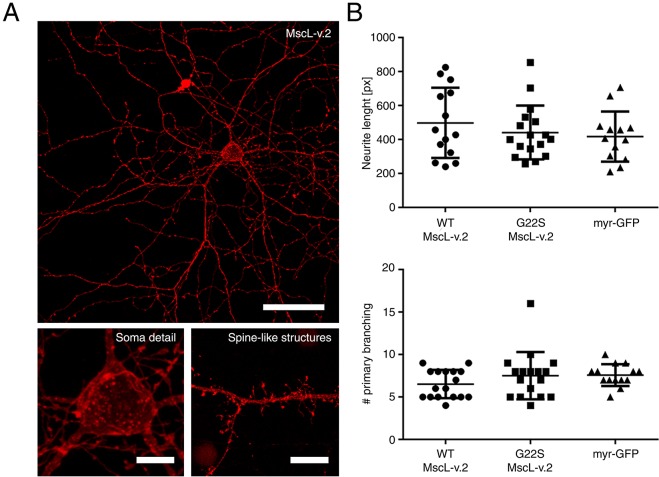
**Morphological evaluation of a neuron expressing the MscL-v.2 construct.** (A) Maximum projection of a confocal *z*-stack of a primary cortical neuron expressing MscL-v.2 fused to tdTomato fluorescent protein (scale bar: 50 µm). The bottom images show the MscL-v.2 fluorescence signal in the soma (left) and spine-like structures (right). Scale bars: 10 µm). (B) The upper panel shows quantification of the neurite length of neurons expressing WT MscL-v.2 (490.30± 55.20, *n*=14), G22S MscL-v.2 (441.50±38.33, *n*=17) or myr-GFP (417.10±41.00, *n*=13). Data are presented in terms of number of pixels and no statistically significant difference was measured. The lower panel shows the quantification of the number of primary neuronal branches calculated for each construct (WT MscL-v.2: 6.53±0.41, *n*=17; G22S MscL-v.2: 7.53±0.68, *n*=17; myr-GFP: 7.57±0.34, *n*=14). Values are reported as mean±s.e.m. and no statistically significant difference was measured.

Importantly, neurons expressing the MscL-v.2 protein showed a good expression level of the channel, even at later (i.e. 20) days *in vitro* (DIV), both in the soma, neurites, and spine-like structures, thus indicating that MscL-v.2 expression was well-tolerated in primary neurons (Fig. 2A; Fig. S1A). However, considering that an enhanced mechanosensitivity could affect neurite growth and branching during network development, we compared the complexity of the dendritic tree in neurons that express the MscL-v.2 with the one in neurons that express only the membrane-targeted GFP.

Furthermore, this analysis was carried out on both wild-type (WT) MscL-v.2 and on a gain-of-function MscL variant bearing a Ser to Gly substitution at position 22 (G22S MscL-v.2) that leads to a lower activation pressure threshold (Yoshimura et al., 1999). As illustrated in Fig. 2B,C, the morphology of neurons expressing either WT or G22S MscL-v.2 did not show any significant alteration in terms of neurite length and number of primary branches compared to the control neurons expressing only myr-GFP. In addition, the complexity of the overall neuronal arborization was unaltered, as determined by the similar number of endpoints between neurons expressing myr-GFP or neurons expressing one of the two versions of MscL-v.2 (Fig. S1B,C). Staining of the synaptic boutons further confirmed the unaltered number of endpoints (see ‘Functional characterization of mechano-sensitized neuronal networks’ and Fig. 4A).

### Electrophysiological characterization of the engineered MscL functionality

**Fig. 3.**
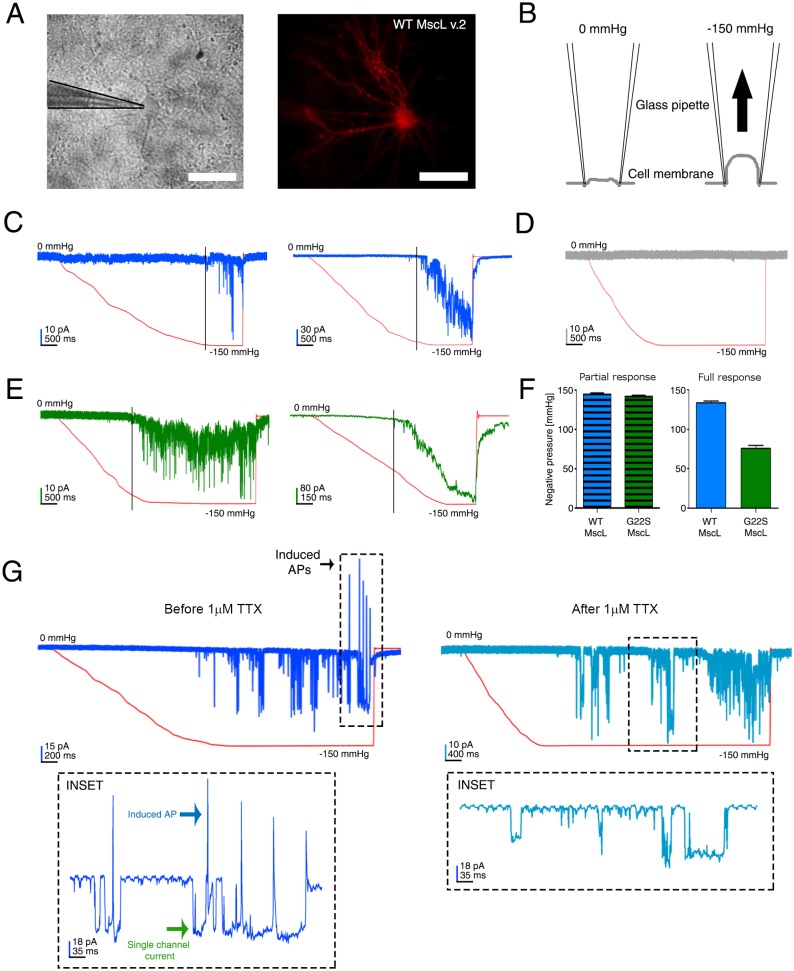
**Electrophysiological characterization of the eMscL expressed in primary cortical neurons.** (A) Bright-field (left) and fluorescence image (right) of a patched cortical neuron (15 DIV) expressing the eMscL construct. The red fluorescence signal is due to the tdTomato fluorescent protein encoded by the eMscL construct. Scale bars: 50 µm. (B) Cartoon indicating the procedure to perform pressure/voltage-clamp recording in cell-attached configuration during pressure-clamp stimulation. Application of a negative pressure induces the cell membrane stretch, which activates the gating of the eMscL. During the stimulation, a command potential of +30 mV was applied and, assuming a resting potential of −70 mV, the estimated applied potential was −100 mV. (C) Traces of the recorded ion currents (blue trace) during pressure stimulation (red trace) of the membrane patch, in a neuron expressing the WT eMscL. On the left, the trace reports a typical example of recorded ionic currents during a partial response. On the right, the current trace of an example of recorded full response. (D) Example of recorded ion current (gray trace) during pressure/voltage-clamp recording of a control neuron expressing only the tdTomato fluorescent protein. (E) Recorded ion currents (green trace) during the pressure stimulation of a neuron expressing the G22S eMscL. On the left, the trace reports a typical example of recorded partial response. On the right, the trace is a representative recording of a full response. (F) Bar graphs show the quantification of the pressure activation threshold required to trigger the WT-induced and G22S eMscL-induced currents. (Left) Quantification of the pressure threshold gating the partial response (145±0.98 mm Hg, *n=*72 stimulation trials, on *n*=19 cells, and 142.50±0.91 mm Hg, *n=*111 stimulation trials, on *n*=24 cells, for WT and G22S channels, respectively). (Right) Quantification of the pressure threshold histogram gating the full response (130±2.36, *n=*48, on *n*=10 cells, and 75.78±3.60, *n=*67 stimulation trials, on *n*=17 cells, for WT and G22S channels, respectively). Values are reported as mean±s.e.m. (G) Example of a recorded ion current trace on a cortical neuron (18 DIV) expressing the G22S channel. The traces correspond to the recorded ion currents on the same neuron before (left) and after (right) incubation with 1 µM TTX (dark blue and light blue traces, respectively). Red curves indicate the application of a negative pressure ramp. The enlarged insets illustrate a detail of the recoded traces in their respective upper panels. (Left) The recorded single-eMscL current (green arrow) and the associated generation of a neuronal action potential (blue arrow) before incubation with TTX. (Right) The sole presence of the eMscL single-channel ion current. Area surrounded by dashed line indicates channel currents with amplitude <50 pA that were associated with the generation of action potentials in both WT and G22S eMscL-expressing neurons.

After confirming the efficient and well-tolerated expression of the MscL-v.2 channel (hence hereafter referred to as eMscL), we verified its functionality and mechanosensitivity through pressure/voltage-clamp recordings in cell-attached configuration. All recordings were performed by patching primary rat cortical neurons at 12-14 DIV (Fig. 3A). Negative pressure was manually applied and set to 150 mm Hg through a customized pressure-clamp system (see Materials and Methods ‘Patch-clamp recordings and pressure-clamp system’), in order to stretch the cell membrane into the patch pipette and, thus, trigger the gating of the eMscL (Fig. 3B). WT and G22S eMscL showed different responses in terms of current amplitude when mechanically stimulated (Fig 3C,E; Fig. S2), indicating the possible presence of distinct sub-conductance states of the channel, as described previously (Cox et al., 2016). Accordingly, we classified the responses into two groups: that of a partial response characterized by bursts of small current events, and that of a full response characterized by higher current amplitude with less noise and a sharp and steep closure following removal of the pressure stimulus. The partial response was often observed during the first cycles of stimulation and was subsequently replaced by a full response. In Fig. 3C,E, representative traces of the induced ion currents upon stimulation of either WT or G22S eMscL (blue or green traces, respectively). Control experiments were carried out with neurons expressing only the tdTomato fluorescence protein, since a specific MscL inhibitor is not available yet. In contrast, in control neurons (*n*=74 stimulation runs on *n*=15 cells) stretch-induced currents were absent (Fig. 3D). These data indicate that currents recorded from eMscL-expressing neurons were due to the specific activity of the engineered channel rather than endogenous expression of other mechanically gated channels or channels belonging to the Piezo family (Tay and Di Carlo, 2017). Finally, we quantified the threshold of pressure activation for both WT and G22S eMscLs (Fig. 3F). Surprisingly, the partial response showed a similar activation threshold for both MscL variants (WT eMscL: 145±0.98 mm Hg, *n*=72 stimulation runs, on *n*=19 cells versus G22S eMscL: 142.50±0.91 mm Hg, *n*=111 stimulation runs, on *n*=24 cells). By contrast, the full response showed a predictable lower activation threshold for the G22S mutant (75.78±3.60 mm Hg, *n*=67 stimulation runs, on *n*=17 cells) when compared to the WT (130±2.36 mm Hg, *n*=48, on *n*=10 cells). Indeed, the partial response might well be due to the interaction of the cell cytoskeleton with the plasma membrane, which counteracts the membrane stretch and the complete opening of the MscL. Likewise, the similar activation threshold measured for the partial response in both WT and G22S expressing cells might reflect the membrane resistance to stretch (Martinac, 2014).

In this regard, for a better understanding of the stretch strain provided on the plasma membrane, we also estimated the bilayer tension corresponding to the measured activation pressure thresholds for the WT and G22S channels (see Materials and Methods ‘Estimating the applied membrane tension’).

Under our experimental conditions, taking in account two values of adhesion energy between cell membrane and glass pipette [i.e. 3.7 mN m^−1^ in case of the homogenous phospholipid membrane (Ursell et al., 2011), and 1.6 mN m^−1^ in case of the neuronal cell membrane (Suchyna et al., 2009)], we estimated tension ranges of 11.6–13.7 mN m^−1^ at a negative pressure of ∼150 mm Hg; and of 6.2–8.3 mN m^−1^ at a negative pressure of ∼70 mm Hg. Both ranges are in line with those previously described for the WT channels and the G22S MscLs (Rosholm et al., 2017).

Once the functional expression of MscLs in neuronal cells was confirmed, we developed an adeno-associated virus (AAV) that expressed G22S eMscL to allow higher expression rates and, again, carried out the patch-clamp experiments to validate the MscL-induced mechano-sensitization of neurons, when the virally expressed G22S eMscL construct is used.

Also, in this case, we measured in cell-attached configuration the thresholds of the activation pressure of partial and full response currents (141±0.48 mm Hg, *n=*65 stimulation trials and 70±0.72 mm Hg, *n=*21 stimulation trials, respectively), and confirmed the previously measured values for the not virally expressed G22S eMscL construct (Fig. 3F).

Moreover, we measured the activation threshold of the G22S eMscL-induced currents in an excised membrane patch (Fig. S3), showing that the activation pressure (67±0.14 mm Hg, *n=*69 stimulation trials) was similar to that of the G22S full response in cell-attached configuration (Fig. 3F). Taking in account these new sets of data, we also confirmed our hypothesis that the partial response, recorded in cell-attached configuration, reflects the action of the cell cytoskeleton counteracting the cell membrane stretch. Indeed, it is important to take in account that, even if MscLs are gated directly by tension along the plasma membrane, the mechanical properties of the membrane might be altered by cytoskeletal proteins and other scaffold proteins linking the cell to the extracellular matrix (Cox et al., 2016).

Next, we performed the same set of experiments with neurons expressing eMscLs at later DIV (15-18 DIV), when the cultured neuronal network is matured and neurons are able to generate spiking activity (Soloperto et al., 2016), in order to investigate the potential for the eMscL to stimulate the generation of neuronal action potentials (APs). Fig. 3G shows a representative trace that was recorded by patching a neuron expressing G22S eMscLs upon application of a negative pressure ramp. The mechanical stimulation was applied on the same cell patch, before and after application of 1 µM tetrodotoxin (TTX), which blocks voltage-gated Na^+^ channel and generation of spontaneous APs. Induced-spike activity was present in neurons expressing both eMscL variants and absent upon treatment with TTX, while the currents induced by eMscL opening were preserved. Interestingly, only channel currents with amplitude <50 pA were associated with the generation of action potentials in both WT and G22S eMscL-expressing neurons (area surrounded by a dashed line in Fig. 3G) (WT eMscL 5 out of 9 cells versus G22S eMscL 9 out of 17 cells). In contrast, eMscL-induced currents with higher amplitudes failed to trigger APs, presumably due to substantial membrane depolarization. Furthermore, we could occasionally detect an increase of the neuronal spiking activity upon mechanical stimulation (Fig. S4), thus indicating the possibility to modulate the neuronal firing rate. Importantly, control cells did not show any spiking activity associated with this level of mechanical stimulation (*n*=15 cells) – as would be expected, given their lack of mechanical response. Thus, we were also able to exclude a direct cell-intrinsic dependence between the applied negative pressure and the increase in neuronal firing rate.

These experimental results illustrate the successful development of an *in-vitro* model efficiently expressing a functional bacterial MscL in mammalian neuronal networks.

### Functional characterization of mechano-sensitized neuronal networks

**Fig. 4.**
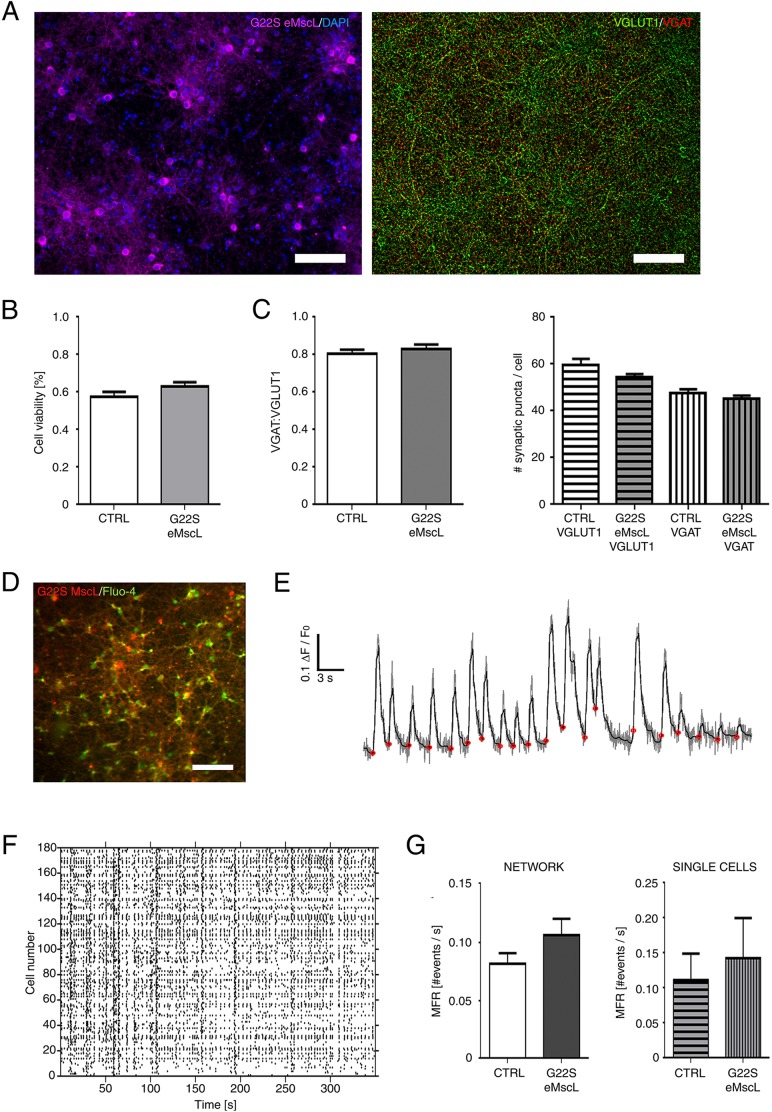
**Functional characterization of cortical neuronal networks expressing the G22S eMscL.** (A) Fluorescence images of a cortical neuronal network (20 DIV) infected with AAV that expresses G22S eMscL. (Left) Fluorescence signal of tdTomato tagged to eMscL (magenta) and DAPI nuclear staining (blue). (Right) Fluorescence image of excitatory and inhibitory synaptic puncta immunostained for the respective markers VGLUT1 (green) and VGAT (red). Scale bars: 100 µm. (B) Bar graph showing the number of viable cells (in percent) in control cultures and in cortical neuronal networks that express G22S channels (57%±3 and 63%±2, respectively). Values are reported as mean±s.e.m. (C, left) Bar graph showing the ratio of VGAT:VGLUT1 synaptic puncta (0.81±0.02 and 0.83±0.03 for control and eMscL-expressing networks, respectively). (Right) Number of VGAT and VGLUT1 synaptic puncta per cell. The average number of synaptic puncta per cell was measured and normalized with respect to the average number of cells per field of view (for control network: VGAT=47.60±1.70 and VGLUT1=59.50±2.75 on 6 fields of view; for G22S-expressing networks: VGAT=64.32±19.25 and VGLUT1=54.50±1.30 on 8 field of views). Values are reported as mean±s.e.m. (D) Fluorescence image showing the field of view of a neuronal network expressing G22S eMscL (red) and the Fluo-4 AM Ca^2+^ indicator (green). Scale bar: 100 µm. (E) Example of a single-neuron ΔF/F_0_ trace of a cortical network (20 DIV). The de-noised trace (black) was superimposed on the raw trace (gray). The red dots indicate the automatically detected onset time of Ca^2+^ fluctuation events (see Materials and Methods). (F) Raster plot of spontaneous Ca^2+^ activity in single cells identified in the field of view of the neuronal network. (G, left) Bar graph showing the mean firing rate (MFR) as number of events per second in control and G22S eMscL-expressing neuronal networks (*n*=10 and 11, respectively). (Right) MFR plot of single cells expressing or not the G22S eMscL within the same neuronal networks (*n*=1380 or 917, respectively). Values are reported as mean±s.e.m.

Since a lower activation pressure of the channel could lead to its potential spontaneous gating during cell reshaping and migration, and considering that mechanical cues play important roles in network maturation, we evaluated the effect of G22S mutant expression in network development and physiology (Fig. 4A). To obtain within the culture the high percentage of eMscL-expressing neurons that is necessary for a network-level study, we infected neuronal cultures with the previously developed AAV expressing G22S eMscL fused to tdTomato fluorescent protein.

First, we compared cell viability and the number of synaptic contacts in control cell cultures and in neuronal networks expressing the eMscL. Analyses were performed on distinct fields of view acquired on each culture (Fig. 4B,C). As illustrated in Fig. 4B, cell viability was preserved in networks expressing eMscL, thus indicating that eMscL membrane expression does not induce cell death (57±3% and 63±2% for control and G22S neuronal networks, respectively). As a further control, we analyzed the viability of only the neurons expressing the G22S eMscL by staining of cell nuclei with propidium iodide dye. We again obtained cell viability of about 59±2% (*n*=9 fields of view), which is consistent with the previous results.

Second, we quantified the number of glutamatergic and GABAergic synapses by immunostaining for the specific markers, the vesicular glutamate transporter 1VGLUT1 (also known as SLC17A7) and the vesicular GABA transporter VGAT (also known as SLC32A1 ), respectively. Both the VGAT:VGLUT1 ratio (0.81±0.02, *n*=6 fields of view for the control networks and 0.83±0.03, *n*=8 fields of view for the eMscL-expressing networks), and the number of excitatory and inhibitory synaptic puncta per cell (Fig. 4C, left and right bar graph, respectively) did not show any significant differences between the control and the eMscL-expressing networks. Therefore, we can conclude that expression of eMscL does not alter the establishment of neuronal connections.

After having verified efficient development of our neuronal networks *in-vitro*, we monitored the spontaneous Ca^2+^ activity after 20 DIV (Fig. 4D) by using the Fluo-4 AM Ca^2+^ indicator. In Fig. 4E, we report a representative trace of the normalized fluorescent Ca^2+^ signal of a single neuron (ΔF/F_0_). The gray line is the raw Ca^2+^ trace, and the superimposed black line is the result of the de-noising algorithm (see Materials and Methods, Ca^2+^ imaging and data analysis). The red dots indicate the onset times of the automatically detected Ca^2+^ events. After extracting and detecting the events of all cells identified within the field of view, we constructed a raster plot of the spontaneous neuronal network activity with single-cell resolution (Fig. 4F). We quantified the mean firing rate (MFR) of neuronal networks expressing the G22S eMscL and compared it to the MFR of control neuronal networks (*n*=12 and 10 cell cultures, respectively). No significant change was detected between the two types of network (Fig. 4G, left panel). As a further control, we also compared the MFRs of single neurons expressing the virally expressed eMscL construct (*n*=917 cells) with those of control cells (*n*=1380 cells) taken from the same network, confirming that the single-cell MFR was unchanged upon eMscL expression (Fig. 4G, right panel). These results show that eMscL expression does not alter neuronal development and integration into a functional network.

## DISCUSSION

The powerful opportunities afforded by cell-type- or tissue-specific sensitization to externally controlled stimuli, are inspiring the development and assessment of novel stimulation methods on the basis of nanotechnology (Rivnay et al., 2017) and/or genetic engineering of cellular sensing elements. Moreover, the development of novel approaches to modulate the activity of neurons and deep brain circuits is pivotal to obtain fundamental understanding of brain (dys)functions, as well as for the design of effective therapeutic strategies to treat neurological disorders. In this regard, the advent of optogenetics has paved the way to the development of versatile experimental approaches that induce the sensitization of neuronal cells through the genetic expression of membrane ion channels with a specific gating response to thermal, chemical or mechanical stimuli, just to mention some recent examples. An alternative route to achieve stimulus sensitization of tissues and cells is offered by the emerging field of nanotechnology (Rivnay et al., 2017). Smart nanoparticles are designed and developed to obtain a localized enhancement of the stimulating field (Carugo et al., 2017; Marino et al., 2017), or a localized transduction of the penetrating signal leading to the modulation of the cellular activities (Marino et al., 2015).

In this context, the exploitation of mechanical signals to remotely affect and control cellular functions is attracting considerable attention in the research community. In fact, a mechanical signal could be easily transmitted deep through dense tissues, thus playing a key role in the modulation of mechano-dependent cellular pathways (Koser et al., 2016).

Here, we show the use of the bacterial MscL to induce the mechano-sensitization of mammalian neuronal cells. Taking into account that MscL directly responds only to membrane tension without requiring any functional interaction with other cellular elements (Cox et al., 2016; Heureaux et al., 2014), we hypothesized that the heterologous expression of such bacterial MS ion channel in primary mammalian cells does not interfere with any intrinsic mechanotransduction pathway of the cell. Therefore, we exploited the opportunity of potentially designing a new mechanotransduction pathway in mammalian cells.

It is worth noting that, thanks to its detailed and broad biophysical characterization (Iscla and Blount, 2012), the MscL could be easily engineered (Liu, 2016). Indeed, well-established procedures to change the mechanosensitivity, channel conductance and gating mechanism of the MscL, are already available. For example, replacement of the Gly residue at position 22 with more hydrophilic/hydrophobic residues, has been shown to decrease/increase the pressure threshold of the channel opening (Yoshimura et al., 1999).

The possibility to control and modify the sensitivity of the channel to mechanical signals is a key feature for the successful development of a mechanogenetic approach. Indeed, considering the analogy with optogenetics, where very few specialized cells present intrinsic sensitivity to light, it has now been established that all cells have some intrinsic mechanism of mechano-sensation, and that the brain itself behaves as a highly mechanosensitive organ (Tyler, 2012). Therefore, to fine-tune the mechanosensitivity of the channel with respect to other cellular sensing elements and to the intensity of the mechanical signal, may represent an effective route to achieve specific activation of selected cellular targets and, thus, overcome the limit of the intrinsic mechanosensitivity of cells. In this regard, two recent studies did exploit the pressure field generated by propagating ultrasound waves and have shown the possibility to achieve spatially resolved neuronal stimulation by either genetically expressing MS channels (Ibsen et al., 2015) or by accurately designing the ultrasound-propagating wavefront (Zhou et al., 2017). Therefore, the development of a cell-type-specific stimulation approach would require both the expression of MS channels with well-tuned sensitivity, and the accurate shaping and calibration of the locally generated ultrasound pressure field. For the above reasons, we designed a viral vector encoding the G22S MscL mutant, as its lower activation threshold might represent a required feature to achieve its selective activation through the use of low-intensity mechanical stimuli that do not stimulate other cellular sensing elements.

Another distinctive property of the MscL is its nominal electrical conductance of 3 nS (Kung et al., 2010), which could be too high for neuronal cells. Nevertheless, the high conductance of the channel might be beneficial in order to accomplish shorter and gentler stimulation of cellular activity, and might be modified accordingly through site-directed mutagenesis assay (Yang et al., 2012). Another characteristic of the MscL that is crucial for its successful functioning *in vivo*, is that it is not ion selective and that it is not straightforward to change the selectivity of such a large pore. Indeed, the channel opening could allow a Ca^2+^ influx that can elicit cellular apoptotic pathways. However, the use of MscLs in mammalian cell culture as a tool for the controlled delivery of bioactive molecules (Doerner et al., 2012) has been previously reported. The authors of this study have shown that cell viability is preserved also for long temporal opening of the channel (in the order of few minutes) when Ca^2+^ is present in the bath solution.

Nevertheless, our results and observations confirm that heterologous expression of functional bacterial MscLs in primary neuronal cultures does not affect cell survival, neuronal network architecture and spontaneous network activity. Moreover, the generation of action potentials associated with channel opening upon application of a calibrated suction pressure, indicates the successful mechano-sensitization of neuronal cells, which could be used to induce and modulate neuronal activity upon mechanical stimulation. In this regard, it is important to highlight that the generation of action potentials was only associated with a partial current response upon mechanical stimulation.

The required suction pressure to induce a partial response was ∼145 mm Hg, which correspond to ∼0.02 MPa. Considering that the range of acoustic pressures that have previously been demonstrated to elicit activity of wild-type neuronal circuits is in the order of ∼0.01–1 MPa (Tufail et al., 2010; Tyler et al., 2008), i.e. well below the typical acoustic pressures inducing thermal or cavitation effects (Dalecki, 2004; Kubanek et al., 2016), we deduce that the activation threshold of the eMscL is appropriate to accomplish its gating through the use of low-intensity ultrasound waves. However, the main challenge in achieving gating of an MS channel by ultrasound pressure waves, originates from a limited understanding of the underlying mechanisms of action, particularly concerning the interaction between low-intensity ultrasound waves and the biological matter (Plaksin et al., 2016), and the corresponding ultrasound field that is required to induce effective membrane strain. This has limited the identification of an optimal delivery of the ultrasound wavefront.

Finally, taking into account the advantages and drawbacks of stimulation approaches, it is worth noting how distinct combinations of core technologies, such as genetic engineering, nanotechnology (Rivnay et al., 2017) and DNA origami, to design ion channels is becoming a common practice to overcome current limitations. As an example, nanopore technologies could be employed to design novel membrane channels *de novo*, utilising a variety of building block materials (e.g. proteins, peptides, DNAs, synthetics and organics) in order to tailor specific pore structures and functions. However, building of novel nanopore architectures is complex, and their assembly and interaction with the cell milieu is not fully predictable (Howorka, 2017). Therefore, the use of biological templates may represent a robust approach for engineering of the pore itself. The coding sequence of our modified bacterial MscL (eMscL) has been optimized for mammalian neuronal expression and trafficking to the plasma membrane by using a neuron-specific promoter and a voltage-gated channel targeting motif. For all the above reasons, we believe that the mammalian engineered eMscL construct represents an important step towards future applications in complex animal models, providing new insights into the mechanobiology of the nervous system (Koser et al., 2016), and paving the way towards the use of eMscL in novel applications of neuro-engineering.

## MATERIALS AND METHODS

### Ethical approval

All procedures involving experimental animals were approved by the institutional IIT Ethic Committee and by the Italian Ministry of Health and Animal Care (Authorization number 110/2014-PR, 19 December 2014). When performing the experiments, we tried to minimize the number of animals that were killed and the potential for nociceptor activation and pain-like sensation, and respected the three Rs (replacement, reduction and refinement) principle, in accordance with the guidelines established by the European Community Council (Directive 2010/63/EU of 22 September 2010).

### Primary neuronal cultures and transfection

Primary neurons were isolated from cortex tissues of Sprague Dawley rats at the embryonic age of 18 days. The female pregnant rats and mice (Charles River Laboratories International) were killed in a CO_2_ chamber and followed by cervical dislocation, before embryos were extracted. Dissected brain tissues were dissociated by enzymatic incubation in 0.25% trypsin (Gibco) and 0.25 mg/ml bovine deoxyribonuclease I (Sigma-Aldrich) for 7 min at 37°C. Before triturating the tissues with a P1000 pipette tip, an equal volume of Dulbecco's modified Eagle’s medium (DMEM, Gibco) supplemented with 10% fetal bovine serum (FBS, Gibco) was added to the suspension to block the trypsin activity. Isolated cortical neurons were counted and plated at a final density of 300 cells/mm^2^ or 500 cells/mm^2^ onto 18 mm glass coverslips.

Before use, glass coverslips were cleaned and pre-coated overnight with 0.1% poly-D-lysine (PDL, Sigma) to enhance cell adhesion.

Neurons were grown in neuronal medium containing Neurobasal medium (NB, Gibco) with 2% B27 supplement (Gibco) and 1% GlutaMAX (Gibco) at 37°C/5% CO_2_ humidified atmosphere. Cultures were maintained up to 25 days *in vitro* (DIV) and fresh medium was added weekly (about 300 µl) to avoid a change in osmolarity due to medium evaporation.

Primary neuronal cells were transfected at 2 DIV with 0.4 µg of MscL plasmid and/or 0.7 µg of myristoylated GFP plasmid (myr-GFP) with Lipofectamine 2000 transfection reagent (Invitrogen). DNA and Lipofectamine at a ratio of 1:1 in a final volume of 300 µl was used for each well. Cells were incubated for 40 min at 37°C/5% CO_2_ with DNA lipofectamine complexes, after which the culture medium was completely removed and replaced with a pre-warmed neuronal medium.

### MscL-v.1 and MscL-v.2 constructs

#### pAAV-hSyn1-MscL-eGFP-v.1 construct

MscL cDNA, kindly provided by Dr Boris Martinac (Victor Chang Cardiac Research Institute, Darlinghurst, Australia), was excised from the pTRE-Tight (Clontech) source plasmid and sub-cloned in-frame with eGFP into pAAV-hSyn1-eGFP vector into *Sal*I and *BamH*I restriction sites.

#### Engineering pAAV-hSyn1-MscL-tdTomato-v.2

To get a more-specific membrane targeting of both WT MscL and G22S MscL, a second-generation of constructs was created as previously described (Gradinaru et al., 2010), by adding the sequence encoding the Kir2.1 endoplasmic reticulum export signal (ERexp) to the C-terminus of our construct. Then, the sequence encoding eGFP was replaced with that encoding tdTomato, as it is known for having a brighter fluorescence signal. The resulting pAAV-hSyn1-MscL-tdTomato-v.2 constructs are referred to as enhanced-MscL (eMscL), i.e. WT eMscL (Addgene ID 107454) and G22S eMscL (Addgene ID 107455).

### Patch-clamp recordings and pressure-clamp system

Primary cortical neurons were plated at a density of 300 cells/mm^2^ onto 18 mm glass coverslip and the voltage-clamp recording was performed in cell-attached configuration between 14 and 20 DIV.

Borosilicate glass capillaries (1.50 mm OD/0.86 mm ID, KF Technology) were pulled by using an horizontal puller (P1000, Sutter Instruments) with a resistance between 6 and 10 MΩ, to generate glass pipettes.

The ‘cell-attached’ experiments were performed by applying a command potential of +30 mV and, assuming a resting potential of −70 mV, the estimated applied potential was −100 mV. Current traces were inverted according to common convention for ‘cell-attached’ recordings. The bath solution contained 140 mM NaCl, 3 mM KCl, 1 mM MgCl_2_, 1 mM CaCl_2_ and 10 mM HEPES pH 7.2; the pipette solution contained 140 mM NaCl, 0.5 mM CaCl_2_, 2 mM EGTA and 10 mM HEPES pH 7.2. EGTA was added to buffer free Ca^2+^. The eMscL-induced currents were amplified through the MultiClamp 700B amplifier (Axon Instruments), and then digitized and recorded with the Digidata 1200A (Axon Instruments) acquisition board. The output current signals were sampled at 25 kHz and filtered using a low-pass filter frequency of 10 kHz.

In order to apply a calibrated negative pressure during the voltage-clamp recording, the setup was equipped with a custom-made pressure sensor system. It comprised a silicon piezoresistive pressure sensor (model MPDX2200DP, Freescale) that generated a linear voltage output directly proportional to the pressure applied in the tubing connected to the patch pipette. The pressure sensor system was connected to a custom-made conditioning circuit and acquired through the MultiClamp 700B amplifier (Molecular Devices). The active conditioning circuit performed amplification, balancing, level shifting and offset compensation of the differential output (temperature and drift compensation) of the pressure sensor, and was based on a double-stage operational amplifier circuitry with onboard offset and gain controls. The output voltage to pressure conversion factor of the overall pressure sensor system was calibrated with a pipette perfusion instrument (2PK+, ALA Scientific Instruments), which was used to apply well-defined negative pressures (in mmHg) to the tubing connected to the patch pipette. During the experiments, the pressure in the tubing was manually applied through a 5 ml Luer-lock syringe, and monitored in real time by using the pCLAMP 10 software (Molecular Devices).

Data acquisition and analysis were controlled using the pCLAMP 10 software package. The pressure activation threshold was determined by observing at which pressure the first evoked current or a relevant change in the trace slope occurred. Data were filtered with a low-pass Bessel filter before the analysis. To verify that the recorded spikes were, indeed, action potentials, we added 1 μM of the neurotoxin tetrodotoxin (TTX) (Tocris Bioscience) to the bath solution and incubated for 5 min to block Na^+^ channels, before applying the negative pressure through the patch pipette.

### Estimating the applied membrane tension

Since the lack of a highly resolved image of the membrane dome in the pipette patch, we estimated the tension that is elicited along the plasma membrane upon mechanical stimulation by applying an equation on the basis of Laplace's law as previously reported (Ursell et al., 2011).

The membrane tension (τ) was estimated by using the equation τ=γ+(rP)/2, where r is the radius of pipette tip (∼1 µm) and P is the applied negative pressure in terms of mN m^−2^.

### Immunostaining and image analysis

For colocalization and morphological analyses, neuronal cells were fixed at 15 DIV; for immunostaining with synaptic markers, cells were fixed at 18-20 DIV.

Neurons were fixed in 4% cold paraformaldehyde (PFA, Sigma-Aldrich) in standard phosphate-buffered saline (PBS, Sigma-Aldrich) for 15 min at room temperature (RT), washed twice in 1×TBS and mounted with ProLong Diamond Antifade mountant (Invitrogen).

For immunostaining, after the fixation protocol was completed, cells were permeabilized with 0.1% Triton X-100 (Sigma-Aldrich) in 1× Tris-buffered saline (TBS) for 5 min at RT, and then blocked with 3% bovine serum albumin (BSA, Sigma-Aldrich) in 1×TBS for 1 h at RT.

Immunostaining was performed by incubating the primary antibody overnight at 4°C and, after a few washing steps in 1×TBS, incubating the secondary antibody for 1 h at RT. During the labeling with secondary antibodies, cells were covered with silver foil to protect the sample from light. Primary antibodies were: guinea pig anti-VGLUT1 (catalog number: 135304, SYSY), rabbit anti-VGAT (catalog number: 131013, SYSY) and neuronal class III beta-tubulin antibody (catalog number: MMS-435P, Covance) diluted 1:500, 1:1000 and 1:250, respectively. Secondary antibodies were: Alexa Fluor 488 goat anti-guinea pig IgG (catalog number: A11073, Life Technologies), and Alexa Fluor 568 goat anti-rabbit IgG (catalog number: A11036, Life Technologies). All secondary antibodies were diluted 1:1000. Primary and secondary antibodies were diluted in 3% BSA in 1×TBS.

Images were acquired on a Leica SP8 confocal microscope (Leica Microsystems) and analyzed with ImageJ, except where otherwise specified.

For neuronal morphology analysis, images were acquired on the DeltaVision Elite microscope (GE Healthcare Life Sciences) using a 20× air objective (PLN 20×/0.4, Olympus). The analysis was performed by running the morphology quantification software NeurphologyJ, an ImageJ plugin, as described by Ho et al. (2011).

Colocalization analysis was performed by using the Coloc2 Image plugin, by following the described procedure (Costes et al., 2004).

The viability plot was calculated as the mean of live cells in percent divided by the total number of cells per field of view, as described by Palazzolo et al. (2017). Apoptotic cells, which are characterized by pyknotic nuclei, were identified by their morphology and counted.

### Production of adeno-associated virus particles

Production of adeno-associated virus (AAV)-eMscL particles was performed in 15-cm culture dishes by using a total amount of 25×10^6^ HEK293T cells (5×10^6^ per dish). Transfections were carried out at 70% cell confluence using a standard Ca^2+^ phosphate-based protocol. The transfected DNAs consisted of a mixture of AAV vector plasmid, AAV serotype 1 and 2 packaging proteins (pRV1 and pH21) at a ratio of 1:1:1, and adenoviral helper (pFdelta6). Seventy-two hours after transfection cells were harvested and AAV particles were extracted by subjecting the cell pellet to three consecutive freeze-thaw cycles and purified through a heparin column (Hitrap Heparin, GE Healthcare).

### Ca^2+^ imaging and data analysis

Primary neuronal cultures were infected with recombinant adeno-associated viral construct (hybrid serotype 1 and 2) encoding G22S eMscL. Primary cultures were infected at 15 DIV by incubating overnight a 1:1000 dilution of the virus stock solution. After incubation, half of the culture medium was replaced with fresh medium.

The infected cell cultures showed a good level of protein expression together with significant Ca^2+^ activity starting from 5 days post infection. Ca^2+^-imaging experiments were assayed between 20 and 25 DIV, after loading cell cultures with the Fluo-4 AM Ca^2+^ indicator (Invitrogen) for 20 min.

Ca^2+^ imaging was performed by using a customized inverted fluorescence microscope that had been integrated with a miniaturized cell incubator (Aviv et al., 2013). The time-lapse Ca^2+^ imaging was performed at a frame rate of 65 Hz through a 10× air objective (NA 0.25, Olympus), 2×2 binning, and EM gain of 120. The acquired time-lapse imaging series (*t*-stack series) were analyzed with a custom written algorithm in MATLAB that has been previously described (Palazzolo et al., 2017).

Briefly, the algorithm computed the standard deviation projection of the *t*-stack and the non-homogeneous background in the projection image was estimated through a morphological opening operation with a disk of arbitrary size (but smaller than the typical dimension of the cell soma), and then subtracted. Successively, the projection image was binarized, and the regions of interest (ROIs) were detected. The fluorescent Ca^2+^ traces of the neurons were then extracted from the *t*-stack by computing the mean fluorescence intensity value within the ROIs previously identified. Subsequently, the raw traces of the neurons were baseline corrected and normalized, to calculate the normalized fluorescent Ca^2+^ signals indicated as ΔF/F_0_ [F= fluorescence intensity in arbitrary units (a.u.)]. The baseline F_0_ of the traces was automatically estimated with a linear diffusion filter that evaluates only the slow varying component of the trace by setting a large time frame (time window duration=30 s). The normalized traces were then smoothed by using the modified Perona-Malik filter (Palazzolo et al., 2017).

On the smoothed traces, Ca^2+^ events were automatically detected by imposing the following conditions: (i) the first derivative in a right interval of the onset overcomes a fixed positive threshold (10^−3^ in case of asynchronous activity, 10^−2^ in case of synchronous activity); (ii) the ΔF between the onset and the offset of an event overcomes a threshold defined as the standard deviation of the difference between the original and the smoothed trace; (iii) the first derivative in a right interval of the event offset is lower than a fixed negative threshold (−10^−4^); and (iv) the time interval between the last time point after the onset with first derivative higher than a fixed threshold and the offset did not reach a fixed width (300 time points).

### Data analysis

Statistical analysis, graphs and plots were generated using GraphPad Prism 6 (GraphPad Software) and MATLAB 2016b (MathWorks). To verify whether our data sets were reflecting normal distribution, the Shapiro-Wilk normality test was carried out. Since the normality distribution was not fulfilled, statistical significance analysis was performed using the nonparametric two-sided Mann–Whitney test (*P*=0.05) and data set are given as mean± standard error of the mean (s.e.m.).

## Supplementary Material

Supplementary information
