# Supplementary material for: Mechano-sensitization of mammalian neuronal networks through expression of the bacterial large-conductance mechanosensitive ion channel
Source: J Cell Sci. 2018 Mar 1;131(5):jcs210393. doi: 10.1242/jcs.210393 (PMC5897719; doi:10.1242/jcs.210393)
Supplement: Supplementary information [file joces-131-210393-s1.pdf]

Supplementary Figures

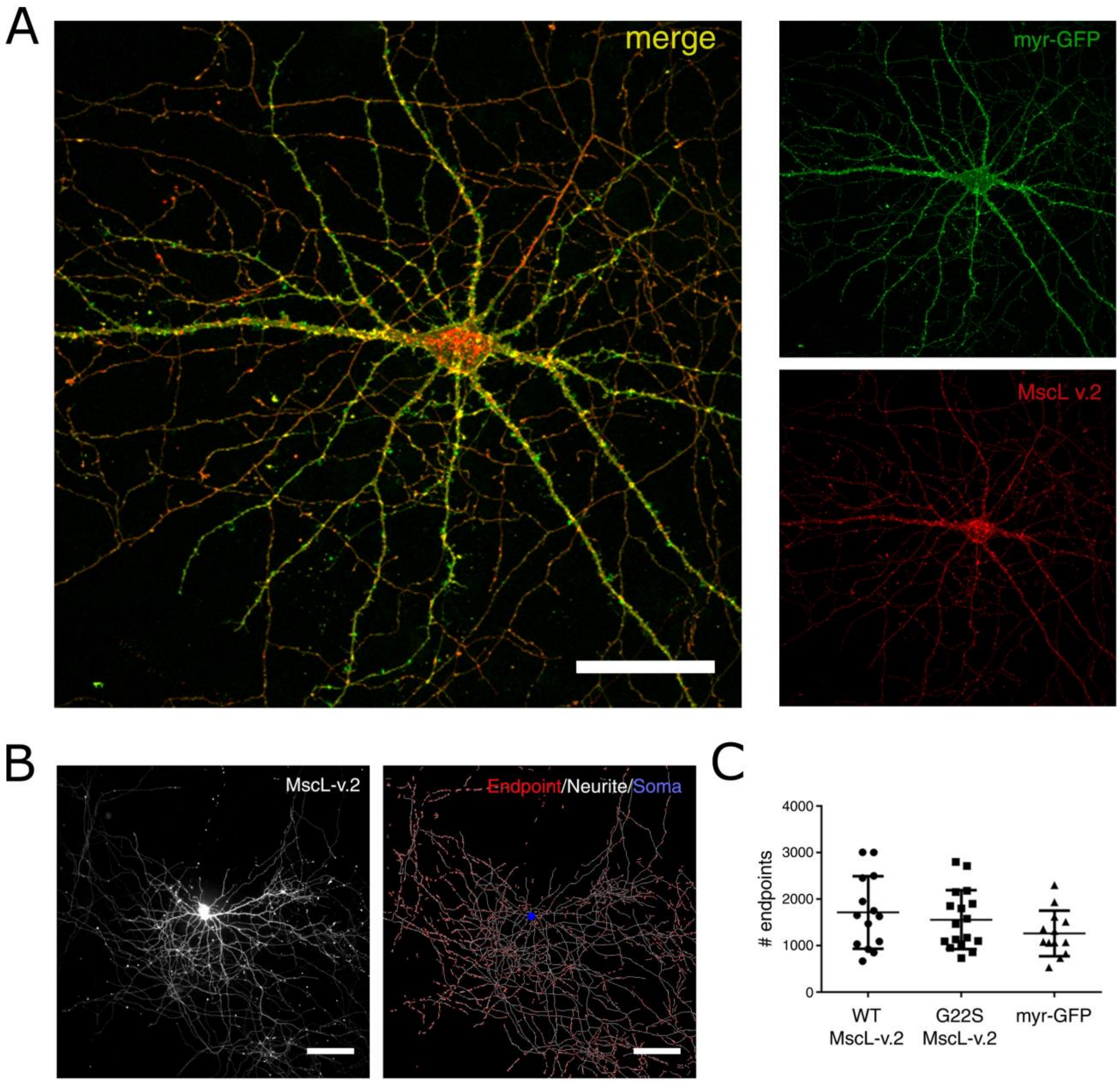

Fig. S1. Cortical neuron expressing the MscL-v.2

A. Cortical primary neuron (20 DIV) expressing the MscL-v.2 (red color), and the myristoylated GFP (green) constructs. The merge of the two fluorescence signals is shown in yellow. Scale bar= 50 μm.

B. On the left, the fluorescence image of a cortical neuron expressing the WT MscL-v.2 construct and, on the right, the detected skeleton of the same neuron is shown. The automatically detected endpoints, neurites, and cell soma are reported in red, white and blue color respectively.

C. Quantification of the number of endpoints detected on neurons expressing the two MscL constructs, and the myr-GFP construct (WT MscL-v.2: 1714±209 endpoints on 14 cells, G22S MscL-v.2: 1559±154 endpoints on 17 cells, myr-GFP: 1262±136 endpoints on 13 cells). Values are reported as mean ± SEM and no statistically significant differences are measured.

Journal of Cell Science • Supplementary information

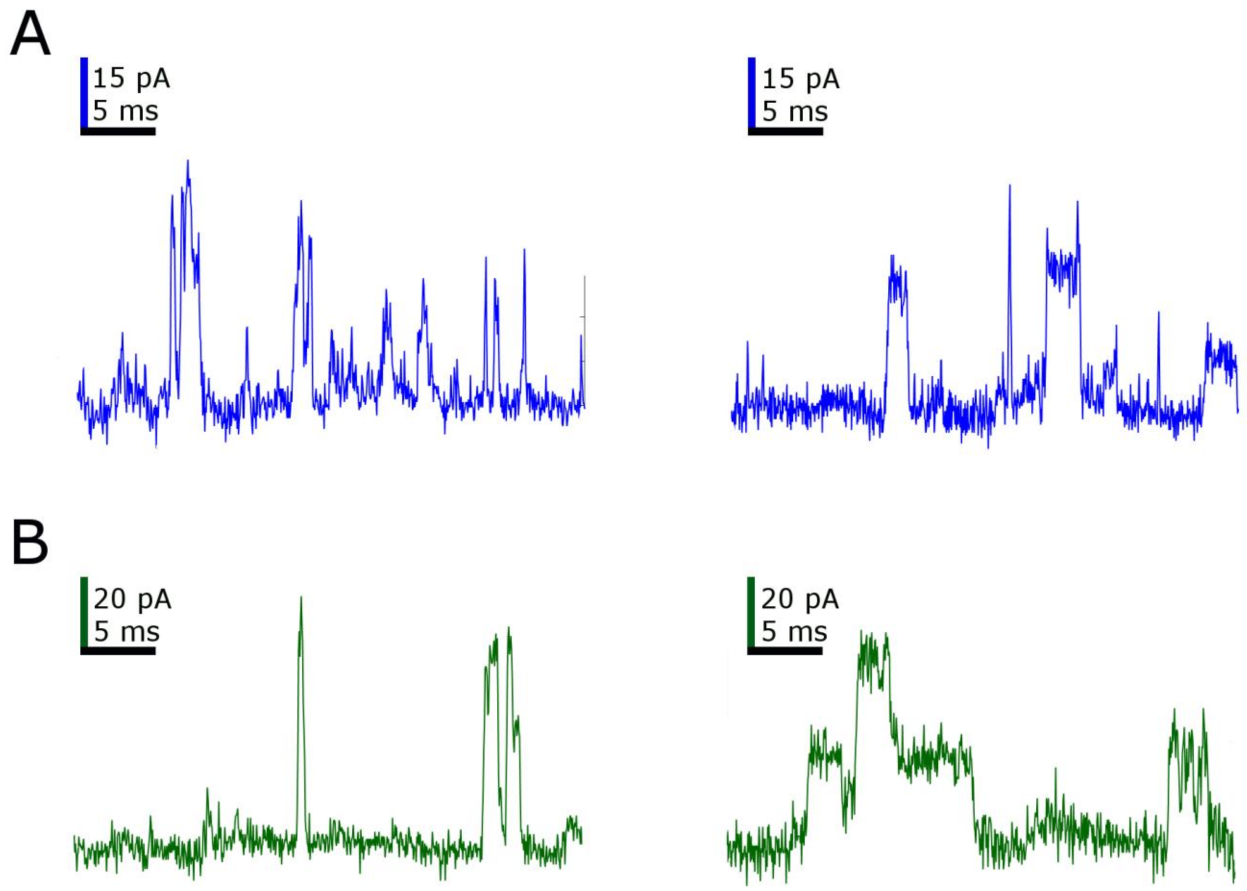

Fig. S2. Expanded view of the WT and G22S eMscL-induced currents in partial and full response.

A. Expanded view of a representative trace of partial response (left panel) and full response (right panel) recorded in WT eMscL expressing neurons in cell-attached configuration.

B. Expanded view of a representative trace of partial response (left panel) and full response (right panel) recorded in G22S eMscL expressing neurons in cell-attached configuration.

Journal of Cell Science • Supplementary information

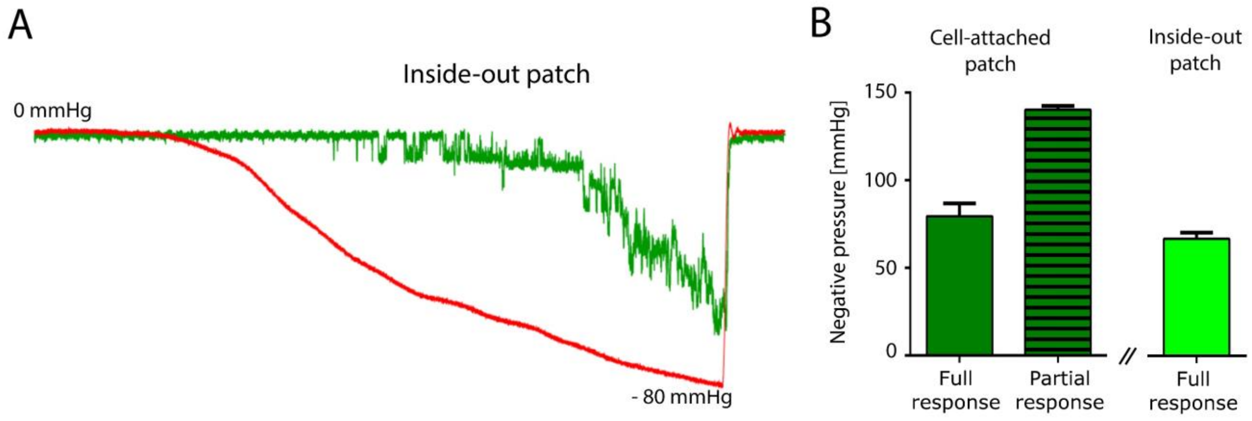

Fig. S3. Characterization of the activation pressure threshold of the virally encoded G22S eMscL construct.

A. Representative trace of the recorded full response (green trace) in excised patch-clamp experiment during the negative pressure stimulation (red trace).

B. Bar plots reporting the quantification of the pressure activation thresholds at which the partial (141±0.48 mmHg, N= 65 stimulation trials) and full (70±0.72 mmHg, N= 21 stimulation trials) current response in cell-attached configuration (green plots), and in excised patch (67±0.14 mmHg, N= 69 stimulation trials) configuration (light green) occur. Values are reported as mean ± SEM.

Journal of Cell Science • Supplementary information

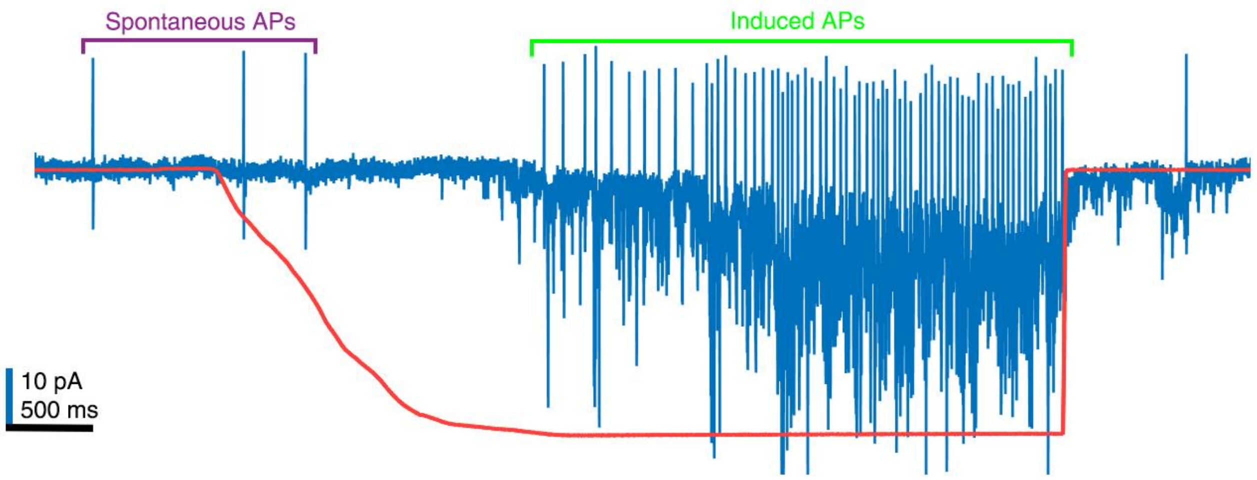

Fig. S4. Mechanical stimulation of neuron expressing the G22S eMscL channel increases its firing rate.

Trace of the recorded ion currents (blue trace) during negative pressure stimulation (red trace) of the membrane patch, in a neuron (18 DIV) expressing the G22S eMscL channel. Violet and green lines respectively highlight the spontaneous and induced APs.

Journal of Cell Science • Supplementary information
